# Inhibition of haematogenous metastasis of colon cancer in mice by a selective COX-2 inhibitor, JTE-522

**DOI:** 10.1038/sj.bjc.6694262

**Published:** 1999-12

**Authors:** S Tomozawa, H Nagawa, N Tsuno, K Hatano, T Osada, J Kitayama, E Sunami, M E Nita, S Ishihara, H Yano, T Tsuruo, Y Shibata, T Muto

**Affiliations:** Departments of Surgical Oncology, Transfusion Medicine, Graduate School of Medical Sciences, Faculty of Medicine, Institute of Molecular and Cellular Biosciences, University of Tokyo, 1-1-1 Yayoi, Bunkyo-ku, Tokyo 113, Japan

**Keywords:** COX-2, colon cancer, metastasis, apoptosis, MMP, selective COX-2 inhibitor

## Abstract

It is proposed that non-steroidal anti-inflammatory drugs (NSAIDs) reduce colorectal tumorigenesis by inhibition of cyclooxygenase (COX). COX is a key enzyme in the conversion of arachidonic acid to prostaglandins and two isoforms of COX have been characterized, COX-1 and COX-2. Multiple studies have shown that COX-2 is expressed at high levels in colorectal tumours and play a role in colorectal tumorigenesis. Recently it has been reported that selective inhibition of COX-2 inhibits colon cancer cell growth. In this study we investigated the effect of a selective COX-2 inhibitor (JTE-522) on haematogenous metastasis of colon cancer. For this purpose, we selected a murine colon cancer cell line, colon-26, that constitutively expresses the COX-2 protein. The subclone P expressed a high level of COX-2 and the subclone 5 expressed a low level. The colon-26 subclones were injected into the tail vein of BALB/c mice. JTE-522 was given intraperitoneally every day from the day prior to cancer cell injection, and the mice were sacrificed 16 days after cell injection. Lung metastases were compared between groups with and without JTE-522. In the mice injected with subclone P, the number of lung metastatic nodules was significantly reduced in the treated group. However, in the mice injected with subclone 5, there was little difference between the control and the treated groups. These results indicate that there may be a direct link between inhibition of haematogenous metastasis of colon cancer and selective inhibition of COX-2, and that selective COX-2 inhibitors may be a novel class of therapeutic agents not only for colorectal tumorigenesis but also for haematogenous metastasis of colon cancer. © 1999 Cancer Research Campaign


*British Journal of Cancer* (1999) **81**(8), 1274–1279
© 1999 Cancer Research Campaign
Article no. bjoc.1999.0841

### Inhibition of haematogenous metastasis of colon cancer in mice by a selective COX-2 inhibitor, JTE-522

**S Tomozawa[1], H Nagawa[1], N Tsuno[1,2], K Hatano[1,2], T Osada[1,2], J Kitayama[1], E Sunami[1], ME Nita[1], S Ishihara[1], H Yano[1,3], T Tsuruo[3], Y Shibata[2] and T Muto[1]**

Departments of [1]Surgical Oncology, [2]Transfusion Medicine, Graduate School of Medical Sciences, Faculty of Medicine, [3]Institute of Molecular and Cellular Biosciences, University of Tokyo, 1-1-1 Yayoi, Bunkyo-ku, Tokyo 113, Japan

**Summary** It is proposed that non-steroidal anti-inflammatory drugs (NSAIDs) reduce colorectal tumorigenesis by inhibition of cyclooxygenase (COX). COX is a key enzyme in the conversion of arachidonic acid to prostaglandins and two isoforms of COX have been characterized, COX-1 and COX-2. Multiple studies have shown that COX-2 is expressed at high levels in colorectal tumours and play a role in colorectal tumorigenesis. Recently it has been reported that selective inhibition of COX-2 inhibits colon cancer cell growth. In this study we investigated the effect of a selective COX-2 inhibitor (JTE-522) on haematogenous metastasis of colon cancer. For this purpose, we selected a murine colon cancer cell line, colon-26, that constitutively expresses the COX-2 protein. The subclone P expressed a high level of COX-2 and the subclone 5 expressed a low level. The colon-26 subclones were injected into the tail vein of BALB/c mice. JTE-522 was given intraperitoneally every day from the day prior to cancer cell injection, and the mice were sacrificed 16 days after cell injection. Lung metastases were compared between groups with and without JTE-522. In the mice injected with subclone P, the number of lung metastatic nodules was significantly reduced in the treated group. However, in the mice injected with subclone 5, there was little difference between the control and the treated groups. These results indicate that there may be a direct link between inhibition of haematogenous metastasis of colon cancer and selective inhibition of COX-2, and that selective COX-2 inhibitors may be a novel class of therapeutic agents not only for colorectal tumorigenesis but also for haematogenous metastasis of colon cancer. © 1999 Cancer Research Campaign

**Keywords**: COX-2; colon cancer; metastasis; apoptosis; MMP; selective COX-2 inhibitor

Several recent studies have reported a 40–50% lower colorectal cancer risk in people who are continuously taking aspirin or other non-steroidal anti-inflammatory drugs (NSAIDs) (Thun et al, 1991; Marnett, 1992; Thun et al, 1993; Giovannucci et al, 1994, 1995; Marnett et al, 1995), suggesting that these drugs may be useful in the chemoprevention of colorectal cancer. The mechanism by which NSAIDs prevent colorectal carcinogenesis is not clearly known, but one possibility involves inhibition of cyclooxygenase (COX). COX is a key enzyme in the conversion of arachidonic acid to prostaglandins, and two isoforms have been identified, namely COX-1 and COX-2. COX-1 is constitutively expressed in most cells and tissues, including normal intestinal and gastric mucosa (Williams and DuBois, 1996). On the other hand, COX-2 is induced by a variety of agents including cytokines, growth factors and tumour promoters; COX-2 expression is elevated in inflammatory cells and sites of inflammation (Kujubu et al, 1991; O'Banion et al, 1991; Jones et al, 1993; DuBois et al, 1994; Xie and Hershman, 1995). Multiple studies have shown that COX-2 is expressed at high levels in 80–90% of colorectal adenocarcinomas (Eberhart et al, 1994; Kargman et al, 1995; Sano et al, 1995), and selective inhibition of COX-2 reduces colorectal tumorigenesis in different models of carcinogenesis (Oshima et al, 1996; Reddy et al, 1996; Kawamori et al, 1998). Thus, COX-2 has been thought to play a causal role in colorectal tumorigenesis.

*Received 3 November 1998*
*Revised 18 March 1999*
*Accepted 19 April 1999*

*Correspondence to:* S Tomozawa

In this study, the inhibitory effect of a selective COX-2 inhibitor, JTE-522, on haematogeneous metastasis was examined in a mouse model of lung metastasis of colorectal cancer.

### MATERIALS AND METHODS

### Preparation of JTE-522

In vivo studies, the selective COX-2 inhibitor, JTE-522 (Matsushita et al, 1997), was suspended in vehicle of 0.5% carboxymethyl cellulose sodium salt (CMC). CMC was obtained from Wako Life Science Reagents (Osaka, Japan). For in vitro studies, JTE-522 was dissolved in dimethyl sulphoxide (DMSO) (Sigma, Watford, UK).

### Animals

Female BALB/c mice were purchased from Oriental Yeast Co., Ltd (Tokyo, Japan). Seven-week-old mice weighing 20 g were used in all experiments.

### Murine colon cancer

Subclones of the murine colon cancer cell line, colon-26, were used. One subclone (clone 5) (Fujimoto-Ouchi et al, 1995) had low COX-2 expression. Another subclone, clone P, established in our laboratory highly expressed COX-2. Both cells were cultured in vitro in RPMI-1640 containing 5% fetal calf serum and 1% antibody (complete medium).

### Western blotting

For the analysis of COX-2 expression, cells were seeded in 100 mm plastic dishes and grown to subconfluence in complete medium. The medium was removed, and cell monolayers were washed three times with phosphate-buffered saline and homogenized in Tris-saline (50 mM Tris-HCl (pH 7.6), 150 mM sodium chloride) containing various protease inhibitors (1 mM EGTA, 0.1 mM diisopropyl fluorophosphate, 0.5 mM phenylmethylsulphonyl fluoride, 1 mg $ml^{-1}$ Nα-p-tosyl-L-lysine chloromethyl ketone, 1 mg $ml^{-1}$ antipain, 0.1 mg $ml^{-1}$ pepstain, 1 mg $ml^{-1}$ of leupeptin). The homogenate was spun at 100 000 rpm for 15 min on a TL-100 rotor (Beckman, CA, USA) and the clear supernatant was used as the cell cytosolic protein fraction. The protein concentration of the supernatant was determined using the Bio-Rad DC Protein Assay (Bio-Rad, CA, USA).

Sodium dodecyl sulphate polyacrylamide gel electrophoresis (SDS–PAGE) was performed as described previously (Hasegawa et al, 1990) using a Laemmli buffer system and 10% polyacrylamide resolving slab gels. Proteins were then transferred electrophoretically to polyvinylidine difluoride (PVDF, Immobilon P; Millipore, Bedford, MA, USA). The PVDF membrane was first equilibrated in transfer buffer (192 mM glycine, 25 mM Tris, 20% methanol, pH 8.3). The cell cytosolic fraction samples were reduced with 2% 2-mercaptoethanol (2-ME). Blotting was carried out at 150 V for 1 h (4°C). After blotting, the remaining reactive sites of the PVDF membrane were blocked by incubating the sheets in TBS containing 3% gelatin. The same buffer containing 10% calf serum (CS) was used to dilute either antisera or purified antibodies. The membrane strips were incubated with the goat anti-COX-2 antibody (Santa Cruz Biotechnology Inc., Santa Cruz, CA, USA) overnight (16–20 h) at ambient temperature on a shaker, washed three times for 15 min and incubated with a 1:500 dilution of biotinylated anti-goat IgG (Vector Laboratories, Inc., Burlingame, CA, USA). After 1 h, the strips were washed an additional three times and then incubated with a freshly prepared avidin–biotin complex (ABCkit, Vector) solution for 30 min. After washing three times, colour development was carried out with a solution of 4-chloro-1-naphthol.

### In vitro proliferation assay

The two subclones of colon-26 were seeded at $8 \times 10^4$ per well in 96-well flat-bottomed plates in triplicate in RPMI-1640 medium containing 0.1% bovine serum albumin (BSA) and allowed to adhere overnight. Then medium containing differing concentrations of JTE-522 diluted in DMSO was added at final concentrations of 0, 10, 25 and 50 µM. After 48 h, the proliferative activity was determined by MTS assay (CellTilter 96TM Non-Radioactive Cell Proliferation Assay, Promega Corporation, Madison, WI, USA), which monitors the number of viable cells. After 4 h in medium containing MTS, the conversion of MTS to formazan was measured in a plate reader at 490 nm. As JTE is used dissolved in DMSO, and different concentrations of DMSO are used for different doses of JTE, the proliferation inhibition curves according to the dose of JTE were expressed as a ratio between the value obtained with JTE and that obtained with DMSO only. $IC_{50}$s were determined from the curves.

### Measurement of apoptosis by DNA fragmentation

Apoptosis was measured by the level of fragmented DNA contained in cell lysates following treatment with JTE-522. The method for measuring fragmented DNA used a commercially available photometric enzyme-immunoassay kit (Cell Death Detection ELISAPLUS; Boehringer-Mannheim, Mannheim, Germany). The immunoassay utilizes mouse monoclonal antibodies directed against DNA and histones, respectively, which allows the determination of mononucleosomes and oligonucleosomes in the soluble fraction of cell lysates. The two subclones of colon-26 were cultured in 10-$cm^2$ dishes in complete medium and allowed to grow to subconfluence. Then the medium was removed and 0.1% BSA–RPMI-1640 containing JTE-522 diluted in DMSO was added at a final concentration of 100 µM (JTE group). To the control group, only DMSO was added. After 24 h of treatment, both floating and attached cells were collected, then lysed and assayed for fragmented DNA according to the protocol specified by the manufacturer. Absorbance was determined by absorption (405–490 nm) using a Bio-Rad microplate reader (Bio-Rad).

### Gelatin zymography assay

To investigate the effect of JTE-522 on the production of matrix metalloproteinases, gelatin zymography was performed as described (Kato et al, 1995), with some modifications. Briefly, the two subclones of colon-26 were treated with JTE-522 (0 and 50 µM) in RPMI-1640 medium containing 0.1% BSA for 24 h. Then cells were harvested by trypsinization, washed 3 times in PBS, and $1 \times 10^7$ cells were suspended in serum-free DMEM: F12 (1:1 ratio) medium and incubated for another 5 h. The cell supernatants were collected, centrifuged to eliminate the contaminating cells, and then concentrated approximately 25 times in a centricon concentrator 10 (Amicon, Inc., Beverly, MA, USA) by centrifugation. The samples were then subjected to SDS-PAGE in 10% acrylamide gels containing 0.1% gelatin. The gels were washed in a 2% Triton X-100 solution for 1 h at room temperature, transfered to the incubation buffer and incubated overnight at room temperature to allow the MMPs to degrade gelatin. The gels were stained for 30 min with Coomassie blue for visualization of the MMPs.

### Murine lung metastasis of colon cancer model

Mice were divided into two groups: control group ($n$ = 6) and JTE group ($n$ = 6). Mice in the JTE group received 0.2 mg JTE-522 suspended in 20 µl 0.5% CMC intraperitoneally (i.p.) and those in the control group received only 20 µl 0.5% CMC i.p., every day from 1 day prior to cancer cell injection. For both groups, a single cell suspension of the colon-26 subclones in Hanks' solution was injected into the tail vein of BALB/c mice ($5 \times 10^4$ cells per mouse). Sixteen days after injection, the mice were sacrificed, the lungs were removed, flushed with India ink, and fixed with Fekete's solution. Lung metastasis was evaluated macroscopically: the number of metastatic nodules was counted and the lungs were weighed.

### Assay of colon cancer cell adhesion to lung

Mice were divided into two groups: control group ($n$ = 8) and JTE group ($n$ = 8). Mice in the JTE group received 0.2 mg JTE-522 suspended in 20 µl 0.5% CMC i.p. and those in the control group received only the vehicle, once a day from the day prior to cancer cell injection.

Clone P cells were labelled with 200 µCi sodium [$^{51}Cr$] chromate (Du, Pont/NEN Research Products)($1 \times 10^7$ cells)

© 1999 Cancer Research Campaign 

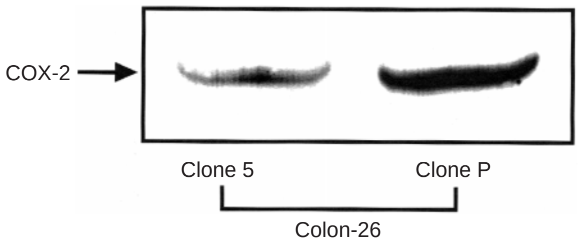

**Figure 1** Western blotting analysis of COX-2 expression levels in colon-26 subclones. The cytosolic protein fraction was obtained by cell homogenization, and after adjustment for protein concentration, the samples were subjected to SDS-PAGE. Bands indicating COX-2 protein were detected at $M_r$ 69 000. Clone P expressed a high level of COX-2 and clone 5 a low level

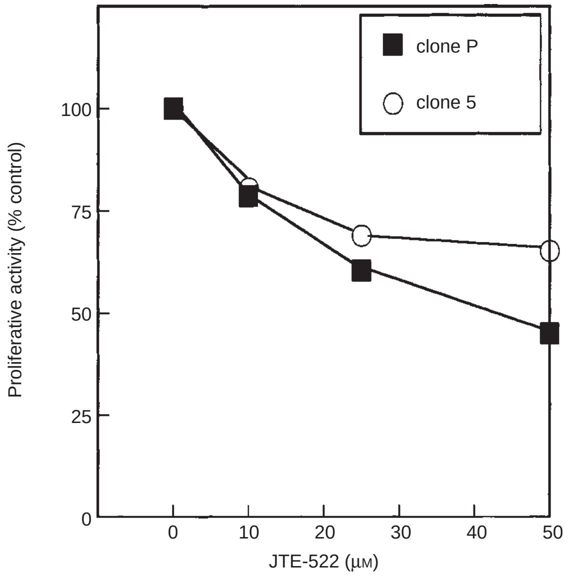

**Figure 2** Proliferation activity (% control) of colon-26 subclones assessed by MTS dehydrogenase activity in cell culture in the presence and absence of various concentrations of JTE-522. Results are expressed as the mean of three different experiments, and the s.d. values are so small that were not expressed. $IC_{50}$ determined from dose–response curves were 41.5 ± 0.6 µM and 150.0 ± 1.7 µm for clone P and 5 respectively. The inhibitory effect of JTE-522 was more pronounced in clone P

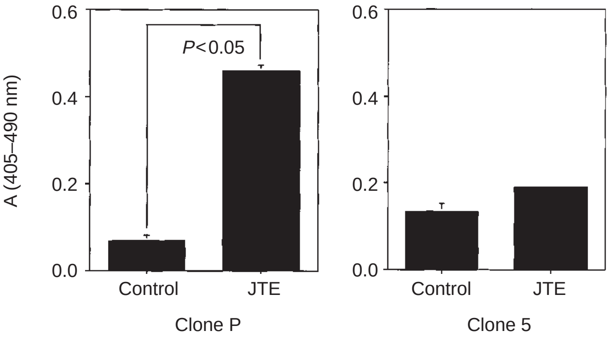

**Figure 3** Detection of apoptosis by measurement of DNA fragmentation in colon-26 subclones after treatment with 100 µM JTE-522 for 24 h. The vertical axis shows the optical density at 405–490 nm, which represents the levels of apoptosis detected in clone P (left) and clone 5 (right) after treatment with JTE-522. Clone P showed an approximately sevenfold increase of apoptotic cells in the treated cells compared to control cells. This difference was statistically significant ($P < 0.01$). In clone 5, JTE-522 treatment caused a small increase in apoptosis, but without statistical significance

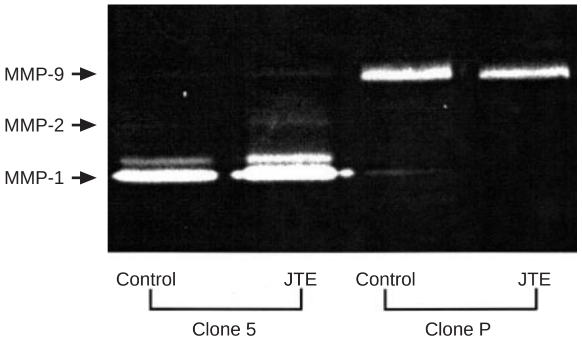

**Figure 4** Gelatin zymography of MMPs secreted into the cell culture medium by colon-26 subclones. Lanes 1 and 3 show the MMPs produced by clones 5 and P, respectively, without JTE treatment and in lanes 2 and 4, following treatment with JTE-522 for 24 h (50 µM). Clone P produced high levels of MMP-9 and small levels of MMP-1. JTE treatment resulted in decreases in the production of both MMPs. Clone 5 produced high levels of MMP-1 and low levels of MMP-9 and MMP-2. JTE-522 treatment did not decrease MMP production, but even resulted in small increases in the production of all MMPs for 1 h, washed three times in complete medium, suspended in Hanks' solution, and injected into the tail vein of the BALB/c mice ($5 \times 10^4$ cells per mouse).

Twenty hours after injection of cancer cells, mice were sacrificed, the lungs removed, and $^{51}Cr$-release from the lungs was measured in a γ-counter.

### Statistical analysis

All results are expressed as mean ± s.d. and were analysed by two-tailed Student's *t*-test. Differences were considered statistically significant at $P < 0.05$.

### RESULTS

### Expression of COX-2 in subclones

Western blotting analysis showed that COX-2 is constitutively expressed in colon-26 cell line. Clone P established in our laboratory expressed a high level of COX-2 whereas clone 5 expressed a relatively low level (Figure 1).

### JTE-522 inhibits colon cancer cell proliferation in vitro

The effect of JTE-522 on colon cancer cell proliferation activity was measured by the MTS method (Figure 2). JTE-522 showed a dose-dependent inhibitory effect on the proliferation of both subclones. However, the growth inhibitory effect on clone P was greater than that for clone 5. $IC_{50}$s were determined from dose–response curves of the JTE-522 concentration versus proliferative activity. $IC_{50}$s were 41.5 ± 0.6 µM and 150.0 ± 1.7 µM for clones P and 5 respectively. These results are consistent with the level of COX-2 expression.

### JTE-522 induces colon cancer cell apoptosis in vitro

Figure 3 shows the effect of JTE-522 (100 µM) on colon cancer cell apoptosis measured by Cell Death Detection ELISAPLUS.

© 1999 Cancer Research Campaign

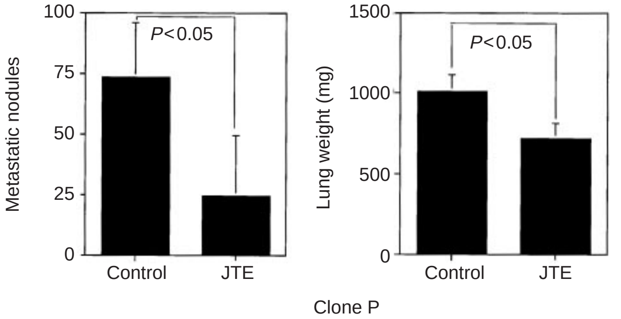

**Figure 5** Number of metastatic nodules (left) and the lung weight (right) in the control and JTE-522-treated groups of mice injected with colon-26 clone P. JTE-522 treatment significantly reduced the number and size of metastatic nodules as well as the lung weight ($P < 0.05$)

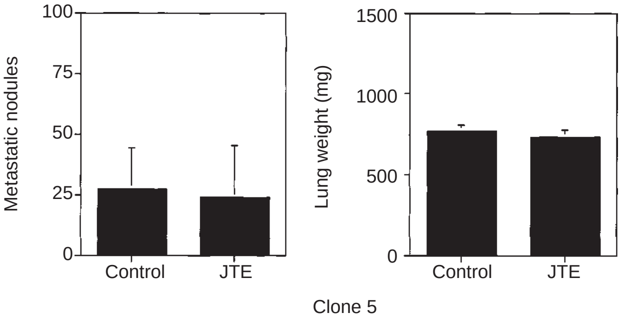

**Figure 6** Number of metastatic nodules (left) and lung weight (right) in the control and JTE-522-treated groups of mice injected with colon-26 clone 5. JTE-522 treatment showed a slight tendency to cause a reduced number of metastatic nodules as well as lung weight, but without statistical significance

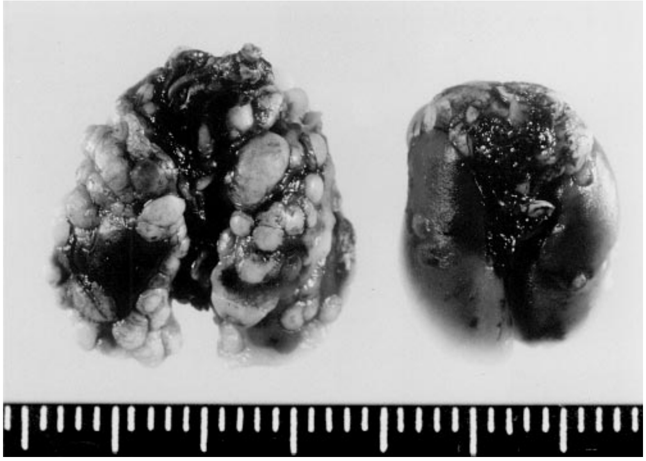

**Figure 7** Representative lungs of mice injected with colon-26 clone P in the control and JTE-522-treated groups. Lungs were injected with India ink and the normal lung parenchyma was stained black, facilitating the visualization of metastatic nodules. In the control group a large number of metastatic nodules were observed with little normal lung parenchyma between them. However, in the treated group, a very small number of nodules were seen

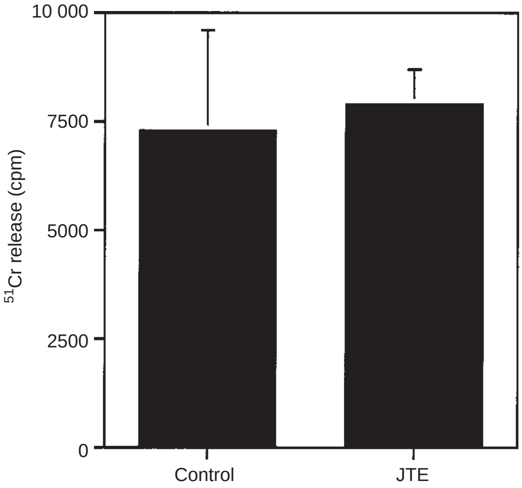

**Figure 8** $^{51}$Cr release from the lungs of the mice injected with $^{51}$Cr-labelled clone P cells measured in a γ-counter (control group $n = 8$ vs JTE group $n = 8$). No significant difference was found ($P > 0.05$)

Treatment of clone P with JTE-522 (100 μM) for 24 h significantly increased the level of fragmented DNA approximately sevenfold (0.068 ± 0.013 in control group, 0.460 ± 0.012 in JTE group, $P < 0.05$). On the other hand, treatment of clone 5 did not significantly induce apoptosis (0.134 ± 0.018 in control group, 0.191 ± 0.005 in JTE group, $P > 0.05$). These results are consistent with the COX-2 level and the results of the proliferation assay.

### JTE-522 reduces the levels of MMPs

As shown in Figure 4, colon-26 clone P produced high levels of MMP-9 (92 kDa) and also low levels of MMP-1 (52 kDa). JTE-522 treatment resulted in decreased production of both MMP-9, and MMP-1 (lanes 3, 4). Differently, clone 5 produced high levels of MMP-1 and low levels of MMP-9 and MMP-2 (72 kDa). JTE-522 treatment had little effect, or even resulted in a small increase in the production of MMPs (lanes 1, 2). These results suggest that JTE-522 treatment induces decreases in the invasive potential of clone P by down-regulating the production of MMPs.

### Lung metastasis

In Figures 5 and 6, the effect of JTE-522 on the number of lung metastatic nodules and the lung weight are shown for both clones. No significant side-effects of JTE-522 administration were observed. Figure 6 shows the representative lungs from mice of the control and treatment groups injected with clone P (control group $n = 6$ vs JTE group $n = 6$). In mice injected with clone P, treatment with JTE-522 significantly reduced the number of metastatic nodules (73.2 ± 26.7 in control group, 24.5 ± 24.7 in JTE group, $P < 0.05$) and the lung weight (1011 ± 102.2 mg in the control group, 717.8 ± 96.8 mg in JTE group, $P < 0.05$) (Figure 5). In contrast, in mice injected with clone 5, there was little difference between the two groups, although the average number of metastatic nodules (27.3 ± 16.8 in control group, 23.8 ± 21.3 in JTE group) and that of lung weight (766.7 ± 41.7 mg in control group, 730.2 ± 43.2 mg in JTE group) were lower in the JTE group (Figure 6). In general, clone 5 had lower metastatic potential than clone P. These results are also consistent with those of the in vitro experiments.

*© 1999 Cancer Research Campaign*

### Effect of JTE-522 on colon cancer cell adhesion to lung in vivo

Figure 8 shows $^{51}Cr$ release from the lungs of the mice injected with $^{51}Cr$-labelled clone P cells (control group $n = 8$ vs JTE group $n = 8$). No significant difference was recognized between the two groups (7284.4 ± 2308.1 cpm in the control group and 7870.5 ± 796.6 cpm in the JTE group).

### DISCUSSION

NSAIDs have been reported to reduce colorectal cancer risk (Thun et al, 1991, 1993; Marnett, 1992, 1995; Giovannucci et al, 1994, 1995) and are suggested to act as cancer-preventive agents, inhibiting both isoforms of cyclooxygenase, COX-1 and COX-2. COX-1 is constitutively expressed in most normal tissues and mediates the synthesis of prostaglandins for normal physiological functions (Williams and DuBois, 1996). On the other hand, COX-2 is not detectable in most tissues but is induced by cytokines, growth factors, oncogenes, serum and tumour promoters (Kujubu et al, 1991; O'Banion et al, 1991; Jones et al, 1993; DuBois et al, 1994; Xie and Hershman, 1995). Recently, several studies have reported that COX-2 is up-regulated in most colorectal adenocarcinomas (Eberhart et al, 1994; Kargman et al, 1995; Sano et al, 1995). In a separate study, SC-58635, a selective inhibitor of COX-2, reduced the formation of aberrant crypt foci and inhibited both of incidence and multiplicity of colon tumours in the intestines of azoxymethane-treated rats (Reddy et al, 1996; Kawamori et al, 1998). A null mutation of COX-2 caused a marked reduction in the number and size of intestinal polyps in a murine model of FAP (familial adenomatous polyposis), $APC^{\triangle 716}$ knockout mice (Oshima et al, 1996). These results indicate that selective COX-2 inhibitor could be a novel agent for the treatment of colorectal tumorigenesis.

Recently, it has been reported that in nude mice, selective inhibition of COX-2 by SC-58125 reduced growth of a human colon cancer cell, HCA-7, expressing high levels of COX-2, but it did not reduce the growth of HCT-116 cells lacking COX-2 protein (Sheng et al, 1997). These results indicate that there may be a relationship between cancer growth and COX-2. Another recent report demonstrated that human colon cancer (Caco-2) cells permanently transfected with a COX-2 expression vector acquired increased invasiveness, showing elevated activation of matrix metalloproteinase-2 (MMP-2) and increased RNA level for membrane-type matrix metalloproteinase-1 (MT-MMP-1) (Tsuji et al, 1997). These results suggest that constitutive expression of COX-2 can increase the metastatic potential of colorectal cancer cells. Therefore, it can be proposed that a selective COX-2 inhibitor may suppress metastasis of colon cancer.

In this study, we investigated the potential of a selective COX-2 inhibitor, JTE-522, as an inhibitor of haematogenous metastasis of colorectal cancer, using a murine model of lung metastasis, and found that JTE-522 inhibited lung metastasis of clone P of colon-26, which expressed a high level of COX-2, and did not have a significant inhibitory effect on clone 5, which expressed a low level.

One of the mechanisms of the anti-metastatic effect of JTE-522 may be inhibition of colon cancer cell growth, because JTE-522 inhibited the proliferation of colon cancer cells in vitro in a dose-dependent manner. Recently, it has been reported that NS-398, another selective COX-2 inhibitor, induced apoptosis and suppressed the proliferation of colon cancer cells (Elder et al, 1997; Hara et al, 1997). In this study, we also demonstrated that JTE-522 induced apoptosis of colon cancer cells, and the induction of apoptosis is probably one of the mechanisms of inhibition of colon cancer cell proliferation. Cyclooxygenase inhibitors are known to induce G1 arrest of cell in the cell cycle (Piazza et al, 1997), and we also confirmed that JTE-522 also induced G1 arrest in colon cancer cell lines (data not shown). Consequently, JTE-522 probably induces apoptosis in cells in the G1 phase, and therefore clone 5 that is a slower grower may be less affected than clone P.

Another of the mechanisms of the anti-metastatic effect of JTE-522 may be reduction of the levels of MMPs, once COX-2 transfection to colon cancer cells increased the production of MMPs (Tsuji et al, 1997), and consequent invasiveness. MMPs are enzymes that are essential for cancer cells to invade and entry into and out of blood or lymphatic vessels (intravasation, extravasation) (Chamber and Matrisian, 1997). JTE-522 treatment of clone P markedly reduced the production of MMPs, while in clone 5 it resulted in slightly increased production.

Haematogenous metastasis of colorectal cancer develops through a complicated process: cancer cells detach from the primary site and reach the vasculature by proteolytic cleavage of the surrounding interstitial tissues. Then the cells reach the secondary sites by adhesion to and transmigration through the endothelial cell layer and invasion of the secondary organ, and they proliferate at secondary sites by induction of angiogenesis (Liotta et al, 1986). Inhibition of lung metastasis by JTE-522 may be the consequence of decreased capacity of cancer cells to extravasate from blood vessels and invade the lung, associated with decreased proliferative activity at the lung due to induction of apoptotic death of cancer cells. We also demonstrated that JTE-522 had little effect on the adhesion of colon cancer cells to endothelial cells, another possible mechanism of inhibition of metastasis.

Tumour growth and metastasis require the development of new vessels, i.e. angiogenesis (Folkman, 1990; Mahadevan and Hart, 1990; Horak et al, 1992). Recently, both stimulatory and inhibitory factors of angiogenesis have been found, but it is still unclear whether there is a relationship between a selective COX-2 inhibitor and angiogenesis of colorectal cancer. Prostaglandin $E_2$, a product of COX, has been reported to enhance angiogenesis in vivo (Ziche et al, 1982), and recently it has been reported that diclofenac (a NSAID), an inhibitor of both COX-1 and COX-2, reduced angiogenesis of colon-26 tumours (Seed et al, 1997), and that in rats a selective COX-2 inhibitor, NS-398, inhibited angiogenesis of sponge granuloma tissues (Majima et al, 1997). In another recent report, COX-2 was reported to modulate the production of angiogenic factors by colon cancer cells, while COX-1 was associated with angiogenesis regulation in endothelial cells (Tsujii et al, 1998). These results suggest that one mechanism by which a selective COX-2 inhibitor inhibits colorectal tumorigenesis and metastasis may be the suppression of angiogenesis. We also found that although COX-2 expression was found in endothelial cells and JTE-522 inhibited their proliferation in in vitro studies, this inhibitory effect was not as marked as in colon-26 cells (data not shown).

We did not investigate the synthesis of prostaglandins by the subclones of colon-26 nor the effect of JTE-522 on prostaglandin synthesis, but there are many reports on the relation between prostaglandin synthesis and COX-2 in colon cancer cells. Sheng et al (1997) reported that SC58125, another selective COX-2

 © 1999 Cancer Research Campaign inhibitor, inhibited prostaglandin $E_2$ production in a human colon cancer cell line, HCA-7, that maintained constitutive COX-2 expression and produced significant prostaglandin $E_2$. Moreover, Tsuji et al (1997) demonstrated an eightfold increase in $PGE_2$ synthesis by COX-2 transfected cells compared to the control Caco-2, a human colon cancer cell line.

In this study, we demonstrated that JTE-522 inhibited metastasis of colon cancer cells that highly expressed COX-2. To our knowledge, this is the first report on the effect of a selective COX-2 inhibitor on cancer metastasis.

The selective COX-2 inhibitor, JTE-522, used in our study is a novel agent that has been recently developed. Consequently, little is known on its exact mechanism of action, and further studies are necessary to investigate its effects in the various steps of the metastatic process.

In conclusion, selective COX-2 inhibitors may be a novel class of therapeutic agents, not only for colorectal tumorigenesis, but also for prevention of haematogenous metastasis of colorectal cancer.

### ACKNOWLEDGEMENTS

We thank Japan Tobacco Co., Ltd (Osaka, Japan) for kindly donating the selective COX-2 inhibitor, JTE-522. We also thank Dr Yutaka Tanaka, Nippon Roche Research Center, for providing the clone 5 of colon-26. This work was supported partly by a Grant-in-Aid for Scientific Research from the Ministry of Education, Sciences, Sports and Culture of Japan and partly by a grant from the Ministry of Health and Walfare of Japan.

### REFERENCES

Chamber AF and Matrysian LM (1997) Changing views of the role of matrix metalloproteinases in metastasis. *J Natl Cancer Inst* **89**: 1260–1270

DuBois RN, Awad J, Morrow J, Roberts LJ and Bishop PR (1994) Regulation of eicosanoid production and mitogenesis in rat intestinal epithelial cells by transforming growth factor and phorbol ester. *J Clin Inves* **93**: 493–498

Eberhart CE, Coffey RJ, Radhika A, Giardiello FM, Ferrenbach S and DuBois RN (1994) Up-regulation of cyclooxygenase 2 gene expression in human colorectal adenomas and adenocarcinomas. *Gastroenterology* **107**: 1183–1188

Elder DJE, Halton DE, Hague A and Paraskeva C (1997) Induction of apoptotic cell death in human colorectal carcinoma cell lines by a cyclooxygenase-2 (COX-2)-selective nonsteroidal anti-inflammatory drug: independence from COX-2 protein expression. *Clin Cancer Res* **3**: 1679–1683

Folkman J (1990) What is the evidence that tumors are angiogenesis dependent? *J Natl Cancer Inst* **82**: 4–6

Form DM and Auerbach R (1983) $PGE_2$ and angiogenesis. *Prog Soc Exp Biol Med* **172**: 214–218

Fujimoto-Ouchi K, Tamura S, Mori K, Tanaka Y and Ishitsuka H (1995) Establishment and characterization of cachexia-inducing and -non-inducing clones of murine colon 26 carcinoma. *Int J Cancer* **61**: 522–528

Giovannucci E, Rimm EB, Stampfer MJ, Colditz GA, Ascherio A and Willett WC (1994) Aspirin use and the risk for colorectal cancer and adenoma in male health professionals. *Ann Intern Med* **121**: 241–246

Giovannucci E, Egan KM, Hunter DJ, Stampfer MJ, Colditz GA, Willett WC and Speizer FE (1995) Aspirin and the risk of colorectal cancer in women. *N Engl J Med* **333**: 609–614

Hara A, Yoshimi N, Niwa M, Ino N and Mori H (1997) Apoptosis induced by NS-398, a selective cyclooxygenase-2 inhibitor, in human colorectal cancer cell lines. *Jpn J Cancer Res* **88**: 600–604

Hasegawa M, Arai T and Ihara Y (1990) Immunochemical evidence that fragments of phosphorylated MAP5 (MAP1B) are bound to neurofibrillary tangles in Alzheimer's disease. *Neuron* **4**: 909–918

Horak ER, Leek R, Klenk N, Lejeune S, Smith K, Stuart N, Greenall M, Stepniewska K and Harris AL (1992) Angiogenesis, assessed by platelet/endothelial cell adhesion molecule antibodies, as an indicator of node metastases and survival in breast cancer. *Lancet* **340**: 1120–1124

Jones DA, Carlton DP, McIntyre TM, Zimmerman GA and Prescott SM (1993) Molecular cloning of human prostaglandin endoperoxide synthase type II and demonstration of expression in response to cytokines. *J Biol Chem* **268**: 9049–9054

Kargman SL, O'Neil GP, Vickers PJ, Evans JF, Mancini JA and Jothy S (1995) Expression of prostaglandin G/H synthase-1 and -2 protein in human colon cancer. *Cancer Res* **55**: 2556–2559

Kato N, Nawa A, Tamakoshi K, Kikkawa F, Suganuma N, Okamoto T, Goto S, Tomoda Y, Hamaguchi M and Nakajima M (1995) Suppression of gelatinase production with decreased invasiveness of choriocarcinoma cells by human recombinant interferon beta. *Am J Obstet Gynecol* **172**: 601–606

Kawamori K, Rao CV, Seibert K and Reddy BSC (1998) Chemopreventive activity of celecoxib, a specific cyclooxygenase-2 inhibitor, against colon carcinogenesis. *Cancer Res* **58**: 409–412

Kujubu DA, Fletcher BS, Varnum BC, Lim RW and Herschmann HR (1991) TIS10, a phorbol ester tumor promoter-inducible mRNA from Swiss 3T3 cells, encodes a novel prostaglandin synthase/cyclooxygenase homologue. *J Biol Chem* **266**: 12866–12872

Liotta LA, Rao CN and Wewer UM (1986) Biochemical interactions with the basement membranes. *Annu Rev Biochem* **55**: 1037–1057

Mahadevan V and Hart IR (1990) Metastasis and angiogenesis. *Acta Oncol* **29**: 97–103

Majima M, Isono M, Ikeda Y, Hayashi I, Hatanaka K, Harada Y, Katsumata O, Yamashita S, Katori M and Yamamoto S (1997) Significant roles of inducible cyclooxygenase (COX)-2 in angiogenesis in rat sponge implants. *Jpn J Pharmacol* **75**: 105–114

Marnett LJ (1992) Aspirin and the potential role of prostaglandins in colon cancer. *Cancer Res* **52**: 5575–5589

Marnett LJ (1995) Aspirin and related nonsteroidal anti-inflammatory drugs as chemopreventive agents against colon cancer. *Prev Med* **24**: 103–106

Matsushita M, Masaki M, Yagi Y, Tanaka T and Wakitani K (1997) Pharmacological profile of JTE-522, a novel prostaglandin H synthase-2 inhibitor, in rats. *Inflamm Res* **46**: 461–466

O'Banion MK, Sadowsky HB, Winn V and Young DA (1991) A serum and glucocorticoid-related 4-kilobase mRNA encodes a cyclooxygenase-related protein. *J Biol Chem* **266**: 23261–23267

Oshima M, Dinchuk JE, Kargman SL, Oshima H, Hancock B, Kwong E, Trzaskos JM, Evans JF and Taketo MM (1996) Suppression of intestinal polyposis in $APC^{\triangle 716}$ knockout mice by inhibition of cyclooxygenase-2 (COX-2). *Cell* **87**: 803–809

Piazza GA, Rahm AK, Finn TS, Fryer BH, Li H, Stoumen AL, Pamukcu R and Ahnen DJ (1997) Apoptosis primarily accounts for the growth-inhibitory properties of sulinduc metabolites and involves a mechanism that is independent of cyclooxygenase inhibition, cell cycle arrest, and p53 induction. *Cancer Res* **57**: 2452–2459

Reddy BS, Rao CV and Seibert K (1996) Evaluation of cyclooxygenase-2 inhibitor for potential chemopreventive properties in colon carcinogenesis. *Cancer Res* **56**: 4566–4569

Sano H, Kawahito Y, Wilder RL, Hashiramoto A, Mukai S, Asai K, Kimura S, Kato H, Kondo M and Hla T (1995) Expression of cyclooxygenase-1 and -2 in human colorectal cancer. *Cancer Res* **55**: 3785–3789

Seed MP, Brown JR, Freemantle CN, Papworth JL, Colville-Nash PR, Willis D, Somerville KW, Asculai S and Willoughby DA (1997) The inhibition of colon-26 adenocarcinoma development and angiogenesis by topical diclofenac in 2.5% hyaluronan. *Cancer Res* **57**: 1625–1629

Sheng H, Shao J, Kirkland SC, Isakson P, Coffey RJ, Morrow J, Beauchamp RJ and DuBois RN (1997) Inhibition of human colon cancer cell growth by selective inhibition of cyclooxygenase-2. *J Clin Invest* **99**: 2254–2259

Thun MJ, Namboodiri MM and Heath CWJ (1991) Aspirin use and reduced risk of fatal colon cancer. *N Engl J Med* **325**: 1593–1596

Thun MJ, Namboodiri MM, Calle EE, Flanders WD and Heath CWJ (1993) Aspirin use and risk of fatal cancer. *Cancer Res* **53**: 1322–1327

Tsuji M, Kawano S and DuBois RN (1997) Cyclooxygenase-2 expression in human colon cancer cells increases metastatic potential. *Proc Natl Acad Sci USA* **94**: 3336–3340

Tsujii M, Kawano S, Tsuji S, Sawaoka H, Hori M and DuBois RN (1998) Cyclooxygenase regulates angiogenesis induced by colon cancer cells. *Cell* **93**: 705–716

Williams CW and DuBois RN (1996) Prostaglandin endoperoxide synthase: why two isoforms? *Am J Physiol* **270**: G393–G400

Xie W and Herschman HR (1995) v-src induces prostaglandin synthase 2 gene expression by activation of the c-Jun N-terminal kinase and the c-Jun transcription factor. *J Biol Chem* **270**: 27622–27628

Ziche M, Jones J and Gullino PM (1982) Role of prostaglandin $E_1$ and copper in angiogenesis. *J Natl Cancer Inst* **69**: 475–482

*© 1999 Cancer Research Campaign*
